# Effectiveness and safety of Danshen injections in treatment of cardiac failure: a network meta-analysis

**DOI:** 10.3389/fphar.2024.1319551

**Published:** 2024-03-13

**Authors:** Yuchen Song, Nan Song, Lianqun Jia, Yupeng Pei

**Affiliations:** ^1^ College of Integrated Chinese and Western Medicine, Liaoning University of Traditional Chinese Medicine, Shenyang, Liaoning, China; ^2^ College of Medical Laboratory, Liaoning University of Traditional Chinese Medicine, Shenyang, Liaoning, China; ^3^ Key Laboratory of Ministry of Education for TCM Viscera-State Theory and Applications, Ministry of Education of China, Liaoning University of Traditional Chinese Medicine, Shenyang, Liaoning, China; ^4^ Benxi Campus Management Committee, Liaoning University of Traditional Chinese Medicine, Shenyang, Liaoning, China

**Keywords:** traditional Chinese medicine, cardiac failure, network meta-analysis, salvia miltiorrhiza injection classes, Chinese medicine injection

## Abstract

**Objective:** The purpose of this network meta-analysis (NMA) was to compare the therapeutic effects of various Danshen (*Salvia miltiorrhiza* Bunge [Lamiaceae; Salviae miltiorrhizae radix et rhizoma]) injections on heart failure to determine the optimal Danshen injection combined with conventional treatment.

**Methods:** 8 databases were searched from the inception of these databases to May 2023 to collect randomized controlled trials (RCTs) on the effectiveness and safety of Danshen injections in the treatment of heart failure. This NMA was performed using Stata 16.0 software and R 4.1.3 software.

**Results:** A total of 24 RCTs involving 2,186 subjects were included. The intervention group received Danshen injections plus conventional treatment, involving the following 7 Danshen injections. The results of the NMA showed that Compound Danshen injection + Common (SUCRA: 79.6%) and Sodium tanshinone ⅡA sulfonate injection + Common (SUCRA: 78.0%) exhibited higher total effective rates. Sodium tanshinone ⅡA sulfonate injection + Common (SUCRA: 94.3%) and Danshen injection + Common (SUCRA: 68.2%) were superior to other traditional Chinese medicines in improving left ventricular ejection fraction (LVEF). Danshen injection + Common (SUCRA: 99.9%) and Shenxiong glucose injection + Common (SUCRA: 77.2%) were the most effective in reducing brain natriuretic peptide (BNP). In addition, compared with conventional treatment, all Danshen injections did not increase the risk of adverse reactions.

**Conclusion:** Current evidence shows that all seven Danshen injections are effective for heart failure. Due to the limited quantity and quality of the included studies, our findings need to be verified by more high-quality studies.

## 1 Introduction

Cardiac failure, also known as heart failure, is a group of clinical syndromes (Zhang et al., 2023) of ventricular filling or ejection ability impairment induced by any heart structural or functional abnormalities. Its main signs include dyspnea, fatigue (limited movement tolerance), and fluid retention (peripheral hematoma and pulmonary congestion). There are about 60 million patients with cardiac failure in the world, with a prevalence rate of approximately 1%–3%. With the aging of the population, the number of such patients is estimated to increase (Adamo et al., 2022) in the next few years. It is pointed out in the *Annual Report on Cardiovascular Health and Diseases in China (2020)* that there are about 8.9 million patients with cardiac failure in China, with a prevalence rate of as high as 1.3% (Wang and Hu, 2020). Cardiac failure mainly occurs in old people, most of whom suffer from more than three comorbidities (Zheng et al., 2022). Cardiac failure may affect the functions of organs and viscera in the whole body. Specifically, insufficient renal vascular perfusion may induce renal dysfunction; long-term hepatic congestion or hypoxia may lead to cardiogenic cirrhosis, and pulmonary congestion may increase the probability of respiratory tract infection. In addition, cardiac failure may also limit the daily activities of patients to affect their quality of life (QOL) and psychological status. From the perspective of Western medicine, primary myocardial injury or myocardial injury caused by some inducements may induce abnormal cardiac load and finally result in cardiac failure. In traditional Chinese medicine (TCM), long-term cardiac obstruction, angina pectoris, or congenital heart disease may induce a shortage and stagnation of heart qi and blood stasis to cause a deficiency of both qi and yin, thus finally resulting in cardiac failure. The standardized Western treatment for chronic heart failure (CHF) is mainly symptomatic treatment in clinical practice, such as enhancement of cardiac function, diuretic therapy, and vascular dilation (Meng, 2018). Specifically, diuretics, RAAS (renin-angiotensin-aldosterone system) inhibitors, *β* receptor antagonists, ACEIs (angiotensin-converting enzyme inhibitors), and digitalis are commonly used. However, high-dose diuretics may reduce the effective circulating blood volume, decrease the cardiac output, and thus aggravate cardiac failure. The reduction of blood volume may also induce reflex sympathetic nerve excitation to aggravate the insufficient perfusion of tissues and organs, thus leading to hepatorenal dysfunction. In addition, some common side effects (e.g., hypotension, renal dysfunction, arrhythmia, etc.) of Western medicine treatment also have a certain impact on clinical decision-making (Wang and Liang, 2018). Recently, Western plus traditional Chinese medicine has shown excellent prospects in cardiac failure. In particular, the effectiveness of Danshen (*Salvia miltiorrhiza* Bunge [Lamiaceae; Salviae miltiorrhizae radix et rhizoma]) drugs is prominent. It has been demonstrated that tanshinone IIA can effectively improve patients’ cardiac function and the levels of vWF and 6-keto-PGF in vascular endothelium (Li et al., 2019b). Therefore, some injections with Danshen as the main ingredient have been widely used in cardiac failure, such as Sodium Tanshinone IIA Sulfonate Injection, Danshenchuanxiongqin Injection, Shenfu Injection, and Shenmai Injection (Gao et al., 2018; Li et al., 2019a; Li et al., 2019b). Shao-mei Wang et al. conducted a randomized controlled trial (RCT) on 120 patients, and found that Shenmai Injection could effectively improve the energy metabolism of patients with cardiac failure (Wang et al., 2020). Many other studies have also demonstrated the effectiveness of Sodium Tanshinone IIA Sulfonate Injection and Shenfu Injection (Wang, 2015; Qiao, 2017; Wang et al., 2019). In addition to monotherapy, Miaomiao Li et al. have also compared the effects of Shenfu Injection + Levosimendan and Levosimendan alone in acute heart failure (AHF). Their study is a single-blind RCT, with 101 patients enrolled, and has revealed that the efficacy of combined medication in AHF is better (Li et al., 2022b).

The Danshen injection treatment group has demonstrated potential in modulating the deposition of myocardial type I and type III collagen, coupled with the regulation of MMP-2 expression. In addition, Danshen injection has shown anti-inflammatory effects by decreasing iNOS and MPO. These findings suggest a significant preventive role for Danshen injection in myocardial fibrosis, cardiac hypertrophy, haemodynamic deterioration, and systolic and diastolic dysfunction associated with heart failure. Consequently, Danshen-based injections emerge as crucial therapeutic agents for heart failure (Wang et al., 2017). However, the existing studies lack a clear elucidation of the efficacy associated with specific Danshen injections (Yuan et al., 2019). In response to this gap in knowledge, this study aims to enhance the available treatment options for heart failure. To achieve this goal, we plan to expand the scope by incorporating a more diverse range of Danshen injections. Furthermore, we aim to conduct a thorough and comprehensive randomized controlled trial, with the overarching objective of establishing a more standardized outcome index. A previous meta-analysis based on the head-to-head comparison at present summarized the clinical results of five types of TCM injections in cardiac failure, including Shenmai Injection, Shenfu Injection, Danhong Injection, Shengmai Injection, and Astragalus Injection. The meta-analysis has proved that Danshen injections, as an auxiliary means of conventional Western medicine treatment (Common), outperform conventional Western medicine treatment alone in total ejection fraction (EF), the relief of cardiac failure symptoms, and the improvement of biochemical indicators (Bai et al., 2018). However, the majority of studies included in that meta-analysis have a small sample size, and the types of drugs included in the studies are limited. Therefore, it is not yet clear which Danshen injection + Common can achieve optimal effectiveness in cardiac failure. To provide more accurate guidance for clinical practitioners to develop optimal treatment strategies for patients, a more comprehensive network meta-analysis (NMA) based on recent RCTs was conducted to identify the most reliable Danshen injection for cardiac failure by integrating as much information as possible through direct and indirect comparisons.

## 2 Method

This NMA was carried out following the preferred information to report for systematic review incorporating NMA (PRISMA-NMA) guidelines (Qiao, 2017) and the Cochrane Collaboration Handbook (Wang et al., 2019). This study was registered in PROSPERO (Registration Number: CRD42023432533). This study included data from previous studies without individual patients. Consequently, no prior ethical approval was required.

### 2.1 Literature search

Two investigators (l.q.J and y. c.S) independently and meticulously conducted systematic searches on databases including PubMed, Web of Science, Cochrane Library, Embase, Sinomed, CNKI, Wanfang Data, and VIP Database. The search spanned from the establishment of these databases to 30 May 2023. The search language was restricted to Chinese and English, and the search was conducted based on subject words + free words. The key subject terms for the search are listed below: “Cardiac Failure,” “Heart Decompensation,” “Myocardial Failure,” “Cardiac decompensation,” “Myocardial failure,” “Traditional Chinese medicine,” “TCM,” “Danshen injection,” “Danhong injection,” “Salvia miltiorrhiza.” The search strategy is shown in Supplementary Table S1 shows the search strategy.

The references in the included papers, systematic reviews, or meta-analyses in this area were also reviewed to avoid missing any eligible studies.

### 2.2 Literature screening

PICOS was used to develop the inclusion criteria: 1) *p* (Population): The study objects were adult patients who suffered from cardiac failure and were above 18 years; 2) I (Intervention): The interventions included Danshen injection + common Western medicine treatment; 3) C (Comparison): The control measure was common Western medicine treatment (including antiplatelet, anticoagulation, thrombolysis, defibrination, improving cerebral circulation, lowering blood pressure, and regulating blood lipid); 4) O (Outcome): The primary outcome indicators included the total EF, Brain Natriuretic Peptide (BNP), and Left Ventricular Ejection Fraction (LVEF); the secondary outcome indicators included 6-min Walking Distance (6MWD), adverse reactions, and N-terminal pro-brain natriuretic peptide (NT-proBNP); 5) S (Study Design): The study type was an RCT.

Exclusion criteria were as follows: 1) Review, case report, clinical trial, study protocol, or conference paper; 2) Animal and *in vitro* studies; 3) Repeated literature and literature of which the full text is not available; 4) Literature in which the outcome indicators cannot be extracted; 5) Letters, replies, etc.; Two reviewers (JLQ and SYC) independently screened the literature according to the above criteria. Any disagreements during the screening would be settled through discussion or by a third reviewer (PYP).

### 2.3 Extracted data and quality evaluation

Two investigators (JLQ and SYC) independently conducted data extraction. The extracted data included: 1) Basic information: Article title, first author, publication date, country/region, *etc.*,; 2) Study characteristics: Interventions, number of study objects, age, and intervention duration of the test group and control group; 3) Key information required to evaluate the risk of bias of the literature; 4) Outcome indicators of the test group and control group.

### 2.4 Risk of bias

The risk of bias (ROB) of the RCTs included in the study would be assessed by two independent reviewers (JLQ and SYC) with the Cochrane tool for risk of bias (ROB2) (Sterne et al., 2019). The tool was used to assess the following five aspects where bias might have occurred: randomization (3 signal problems), deviation from established interventions (7 signal problems), missing outcome data (6 signal problems), outcome measurement (5 signal problems), and selectively reported results (3 signal problems). The risk of bias for each domain was categorised into three levels based on the reviewers’ answers to each signal question: “low risk of bias”, “some concerns” and “high risk of bias.” If the risk of bias evaluation results for all areas were “low risk,” then the overall risk of bias was “low risk.” If the risk of bias evaluation results for some areas were “some risk” with no “high risk” area, then the overall risk of bias was “some risk.” However, as soon as the risk of bias evaluation results for one area were “high risk,” then the overall risk of bias was “high risk.” Disagreements in the literature were resolved through discussion or seeking advice from a third investigator, and the evaluation results were presented in a ROB plot.

### 2.5 Data integration and statistical analysis

The primary outcome indicators included the total EF, LVEF, and BNP. The secondary outcome indicators included adverse reactions and 6MWD.

The gemtc program package (V 1.0-1) in R (V 4.1.3) and the JAGS software were employed to conduct the NMA based on the Bayesian framework by the Markov Chain Monte Carlo (MCMC) technique. Following established procedures, we assessed the validity of assumptions related to homogeneity, similarity, and consistency. Four Markov chains were used for simulation analysis, with an initial value of 2.5 and a refined iteration step size of 1. 50,000 iterations were pre-simulated for annealing, and 20,000 iterations were to achieve model convergence. A pivotal assumption is that the analyzed network is consistent, devoid of conflicts between direct and indirect evidence (Dias et al., 2013). Model fitting and global consistency were compared based on the Deviance Information Criterion (DIC). If the absolute value of DIC for consistency and inconsistency was less than 5, a consistency model would be used for modeling. Using the gemtc package (V 1.0-1) and JAGS software, we created a network plot with the network function in R (V 4.1.3). The network map function is used to generate a network plot in Stata. Additionally, a heterogeneity test was conducted using the mtc. anohe function. If I^2^ of the two interventions was greater than 50%, it indicated that the combined heterogeneity for the two interventions was large. If there was a closed loop in a network, the local consistency would be analyzed by the node-splitting technique.

The relative risk ratio (RR) was expressed for dichotomous data. The weighted mean difference (WMD) was expressed for continuous data. The RR, WMD, and 95% confidence interval (CI) were obtained and summarized to estimate the differences among interventions. The analysis results included the network relation diagram, ranking of cumulative probability, league table, and “correction-comparison” funnel plot of each outcome indicator. The surface under the cumulative ranking curve (SUCRA), which indicated the cumulative ranking probability, was used to rank the interventions. The closer the value was to 100%, the better the intervention was. The Pairwise and Network Meta-Analysis was completed with the Stata 16.0 and R software (V 4.1.3).

## 3 Results

### 3.1 Screened literature and flow chart

**FIGURE 1:**
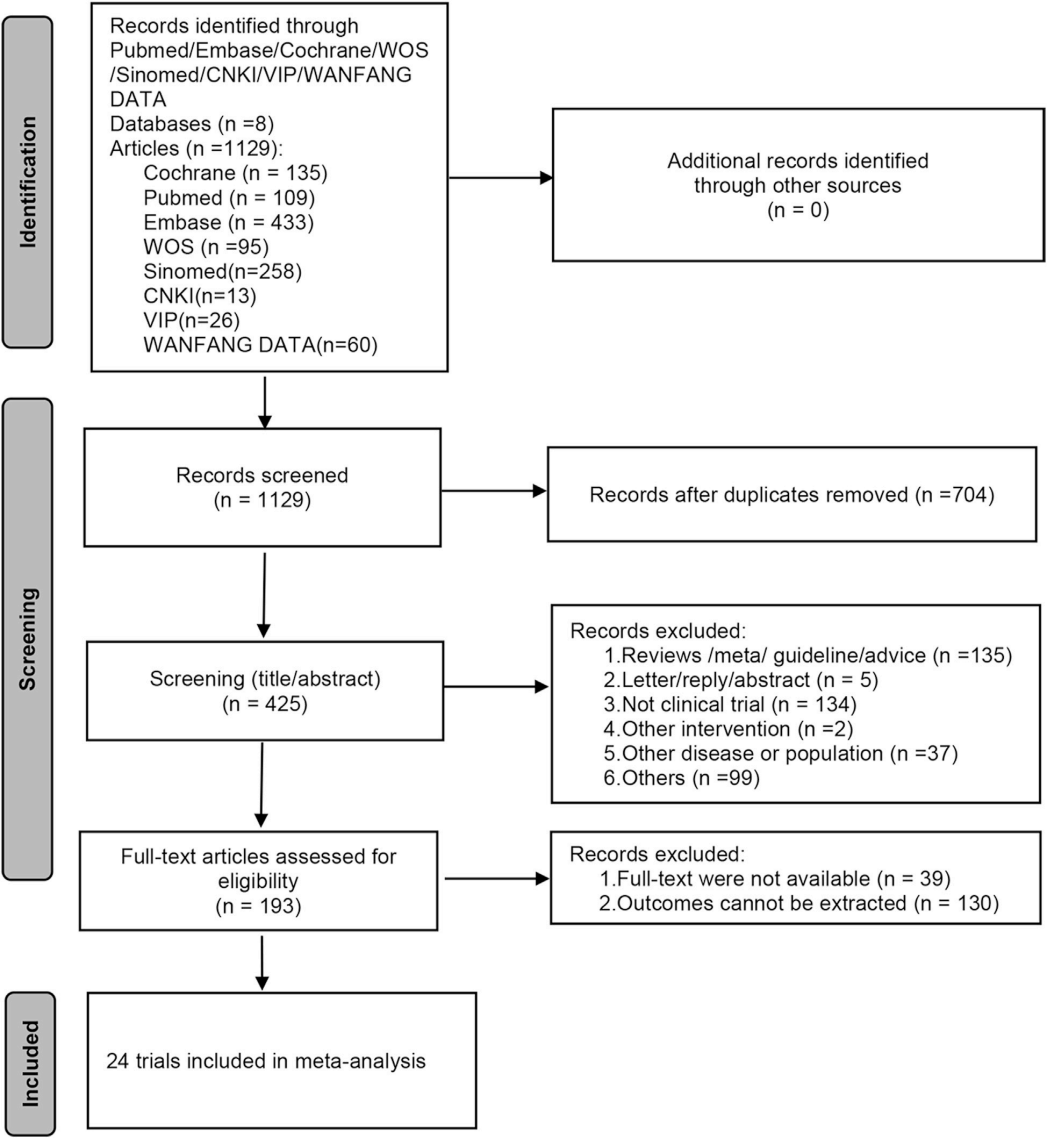
The PRISMA flow chart of the literature search and selection.

In total, 1,129 papers were acquired from the initial database search, and no other study was found in the scanning of references. After eliminating duplicates, a total of 425 papers underwent screening based on titles and abstracts. Among them, 232 papers were excluded as they did not conform to the inclusion criteria. The full texts of 193 papers were meticulously reviewed to select eligible studies, strictly adhering to the predefined inclusion and exclusion criteria. Finally, 24 studies were incorporated into the meta-analysis (Li, 2011; Hu, 2012; Tang, 2013; Hu, 2015; Wang and Gao, 2015; Zeng et al., 2015; Zhang et al., 2015; Jiang et al., 2016; Li, 2016; Lyu et al., 2016; Qin and Jiang, 2016; Xian et al., 2016; Qiao, 2017; Yao, 2018; Shang and Lyu, 2019; Wang et al., 2019; Wang et al., 2020; Li et al., 2022b). The literature screening process is shown in Figure 1.

### 3.2 Basic characteristics of included studies

Seven interventions were involved in our study, including Shengmai Injection + Common (SM + Common), Shenxiong Glucose Injection + Common, Sodium Tanshinone IIA Sulfonate Injection + Common, Danshen injection + Common, Compound Danshen injection + Common, Shenfu Injection + Common, and Danshenchuanxiongqin Injection.

A total of 24 papers were included, in which 2,186 patients were involved, including 1,218 males and 968 females. The number of subjects in the included literature ranged from 18 to 114, and around 50 for most studies. The majority of subjects were middle-aged and older patients (48–84 years), with a mean age of about 60 years. All the included studies were RCTs completed in China. Regarding the evaluation criteria for total EF, the New York Heart Association (NYHA) Cardiac Function Classification Criteria was used in five studies (Clare, 2007), the Short-form 36 (SF-36) Heart Survey Score in one study, TCM Syndrome Score in four studies, *Guidelines for Clinical Studies of New Drugs of Traditional Chinese Medicine* in one study (Qiao, 2017), *Guidelines for Clinical Studies of New Drugs of Traditional Chinese Medicine* in one study (Li, 2011; Jiang et al., 2016), the diagnostic criteria for pulmonary heart disease concluded at the third professional conference on pulmonary heart disease (Zhang, 2009) in one study (Tang, 2013), the definition of EF and markedly EF described in the *Practical Journal of Cardiac Cerebral Pneumal and Vascular Disease* in one study (Hu, 2012), and the result was calculated according to “total EF = markedly EF + EF (Zhuang, 2010).” Additionally, the evaluation criteria were not specified in 11 studies. The basic statistics of the included studies are presented in Table 1.

**TABLE 1:** Characteristics of the included studies.

First author	Publication year	Country	Study design	Sample size		Sex (male/female)		Age mean ± SD or median (IQR) or Median [range] or range		Disease duration (year)		Treatment		Treatment Duration (days)	Follow up time	Complication	Main outcomes
				Case	Control	Case	Control	Case	Control	Case	Control	Case	Control				
Li et al.	2022	China	RCT	51	50	39/11	36/15	73.00 (69.00, 80.00)	73.00 (67.00, 80.00)	NM	NM	SFI + Common	Common	24 h	NM	Hypotension	BNP
Wang et al.	2019	China	RCT	80	80	42/32	48/22	68.58 ± 8.42	68.14 ± 8.73	NM	NM	SFI + Common	Common	7 ± 1	28 ± 3 days	NM	6MWD,LVEF,BAD
Wang et al.	2020	China	RCT	37	39	23/14	24/15	71.27 ± 11.25	69.74 ± 14.06	NM	NM	SMI + Common	Common	7	NM	NM	BNP
Li et al.	2018	China	RCT	62	62	42/20	44/18	61.24 ± 6.45	60.54 ± 6.21	10.32 ± 1.24	10.18 ± 1.32	SF + Common	Common	14	NM	NM	LVEF
Xian et al.	2016	China	RCT	114	114	71/43	66/48	68.95 ± 9.91	68.12 ± 8.88	NM	NM	SMI + Common	Common	7	NM	NM	BAD,LVEF,BNP,6MWD
Xin et al.	2012	China	RCT	28	28	15/13	16/12	58.9 ± 8.7	59.6 ± 9.2	NM	NM	SMI + Common	Common	7	NM	Coronary heart disease	LVEF, EF
Huang et al.	2022	China	RCT	40	40	22/18	21/19	73.7 ± 8.6	73.9 ± 7.1	95.4 ± 40.0 months	100.9 ± 47.6 months	SXG + Common	Common	14	NM	NM	BNP,LVEF,6MWD
Ou et al.	2019	China	RCT	38	30	23/15	17/13	63.97 ± 13.33	62.82 ± 10.63	10.04 ± 2.76	9.94 ± 2.31	SXG + Common	Common	7	NM	Hypertension	6MWD,LVEF,BNP,EF
Yao et al.	2018	China	RCT	24	24	14/10	16/8	66.4 ± 2.8	67.2 ± 3.1	11.1 ± 3.8	11.8 ± 4.1	SXG + Common	Common	14	NM	NM	BNP,EF
Hu et al	2015	China	RCT	38	30	22/16	16/14	67.1 ± 3.8	66.3 ± 3.2	12.2 ± 5.1	12.0 ± 4.7	SXG + Common	Common	14	NM	NM	EF
Zhang et.al	2015	China	RCT	38	38	34/4	36/2	68.1 ± 7.5	68.5 ± 7.0	NM	NM	SXG + Common	Common	14	NM	COPD	EF
Li et al.	2016	China	RCT	60	60	33/27	36/24	62	60	13	14	DSCXQ + Common	Common	14	NM	Chronic pulmonary heart disease	EF,
Jiang et al.	2016	China	RCT	43	40	NM	NM	NM	NM	NM	NM	STIIAS + Common	Common	14	NM	NM	EF,BNP
Qiao et al.	2017	China	RCT	30	30	18/12	17/13	63.5 ± 20.5	62.4 ± 17.6	2–25	3–26	STIIAS + Common	Common	14	NM	NM	LVEF,EF,BNP
Qin et al.	2016	China	RCT	40	40	23/17	24/16	62. ± 13.4	59.5 ± 11.2	NM	NM	SMI + Common	Common	15	NM	Hypertension	LVEF,EF,BNP
Shang et al.	2016	China	RCT	64	64	35/29	37/27	64.74 ± 8.23	64.62 ± 8.21	NM	NM	DS + Common	Common	14	NM	NM	EF,LVEF
Lv et al.	2016	China	RCT	29	29	16/13	17/12	48.21 ± 7.52	49.12 ± 7.18	12.21 ± 1.83 months	12.32 ± 1.88months	STIIAS + Common	Common	7	NM	NM	BAD,LVEF,BNP
Zeng et al.	2015	China	RCT	49	49	26/23	28/21	40.9 ± 11.2	40.5 ± 11.7	36.2 ± 7.37 days	35.4 ± 7.50 days	DS + Common	Common	21	NM	Hypertensive heart disease	BAD,LVEF,EF,6MWD
Wang et al.	2015	China	RCT	36	36	21/15	20/16	64.8 ± 8.1	65.1 ± 7.5	4.3 ± 0.5	4.1 ± 0.6	CDS + Common	Common	28	NM	End-stage renal disease	EF,LVEF,6MWD
Cao et al.	2015	China	RCT	18	18	21/15		66.3 ± 8.6		NM	NM	CDS + Common	Common	5–7	NM	NM	BAD,EF,LVEF
Tang et al.	2013	China	RCT	40	40	20/20	22/18	65.9 ± 4.5	67.5 ± 3.8	13.2 ± 5.2	12.6 ± 4.3	DS + Common	Common	15	NM	Pulmonary heart disease	EF
Yan et al.	2012	China	RCT	34	34	19/15	18/16	67	65	NM	NM	STIIAS + Common	Common	14	NM	Chronic pulmonary heart disease	EF,LVEF
Hu et al.	2012	China	RCT	50	50	53/47		61.2 ± 3.5		NM	NM	DS + Common	Common	14	NM	NM	EF,LVEF
Li et al.	2011	China	RCT	59	59	30/29	29/30	67.4 ± 6.7	66.8 ± 7.4	NM	NM	DS + Common	Common	21	NM	NM	LVEF

Notes: Data are expressed as mean SD, median (IQR) or range. NM, not mentioned; SFI, Shenfu injection; SMI, Shengmai injection; SXG, Shenxiong glucose injection; STIIAS, Sodium tanshinone ⅡA sulfonate injection; DS, Danshen injection; CDS, Compound Danshen injection; SF, Shenfu injection; DSCXQ, Danshenchuanxiongqin injection. Outcomes: BNP, brain natriuretic peptide; 6MWD, 6-Min Walking Distance; LVEF, left ventricular ejection fraction; EF, efficiency; BAD.

### 3.3 Risk of bias

**FIGURE 2:**
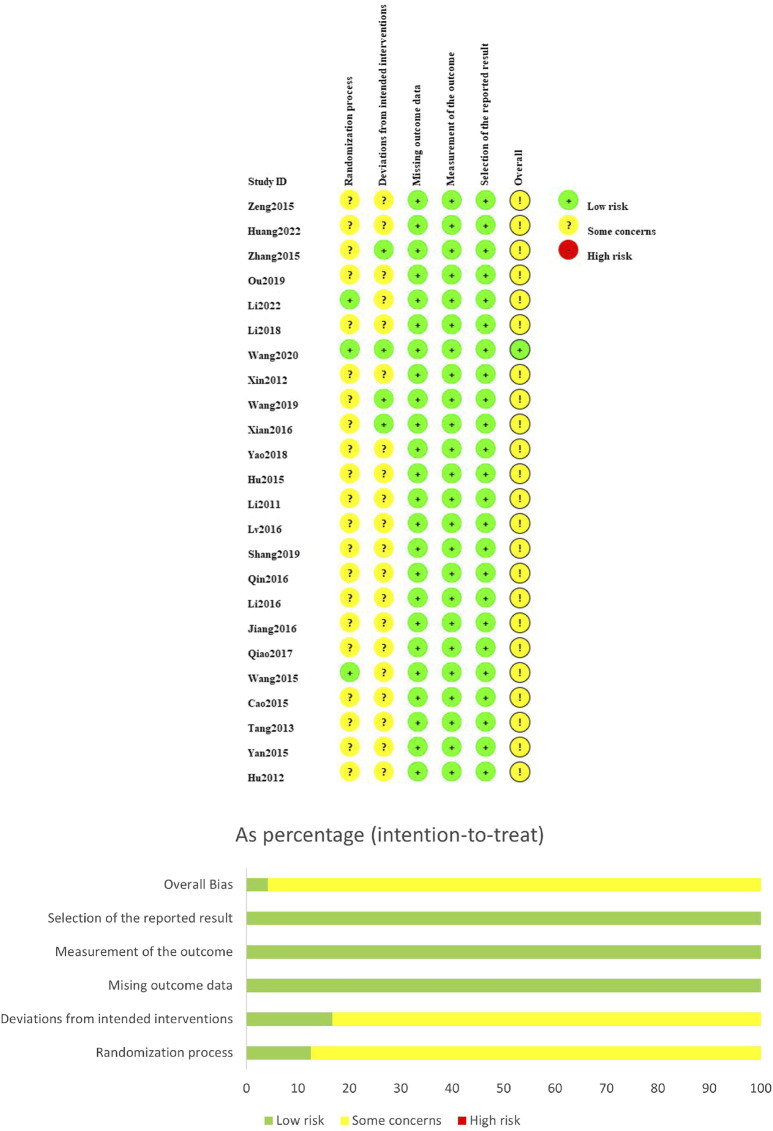
Summary results on risk of bias (using RoB2) of including RCTs.

The ROB assessment result of the 24 included papers is illustrated in Figure 2. For the ROB generated in randomization, 21 studies were assessed as having a potential risk due to the absence of random allocation or allocation concealmen. Conversely, the remaining 3 studies were appraised as having a low risk. Regarding the indicator of deviations from intended interventions, 4 studies were considered as low-risk, while the other 20 studies were evaluated as having some concerns as they did not provide specific information about blind techniques. In the missing outcome data, measurement of the outcome, and selective reporting, the 24 RCTs were evaluated as low-risk as they all reported complete data and measured the outcome indicators in a standardized manner, and there was no risk of selective reporting. Overall, 23 studies were evaluated as having a possible risk, and 1 study was evaluated as low-risk.

### 3.4 NMA results

#### 3.4.1 Network evidence map

**FIGURE 3:**
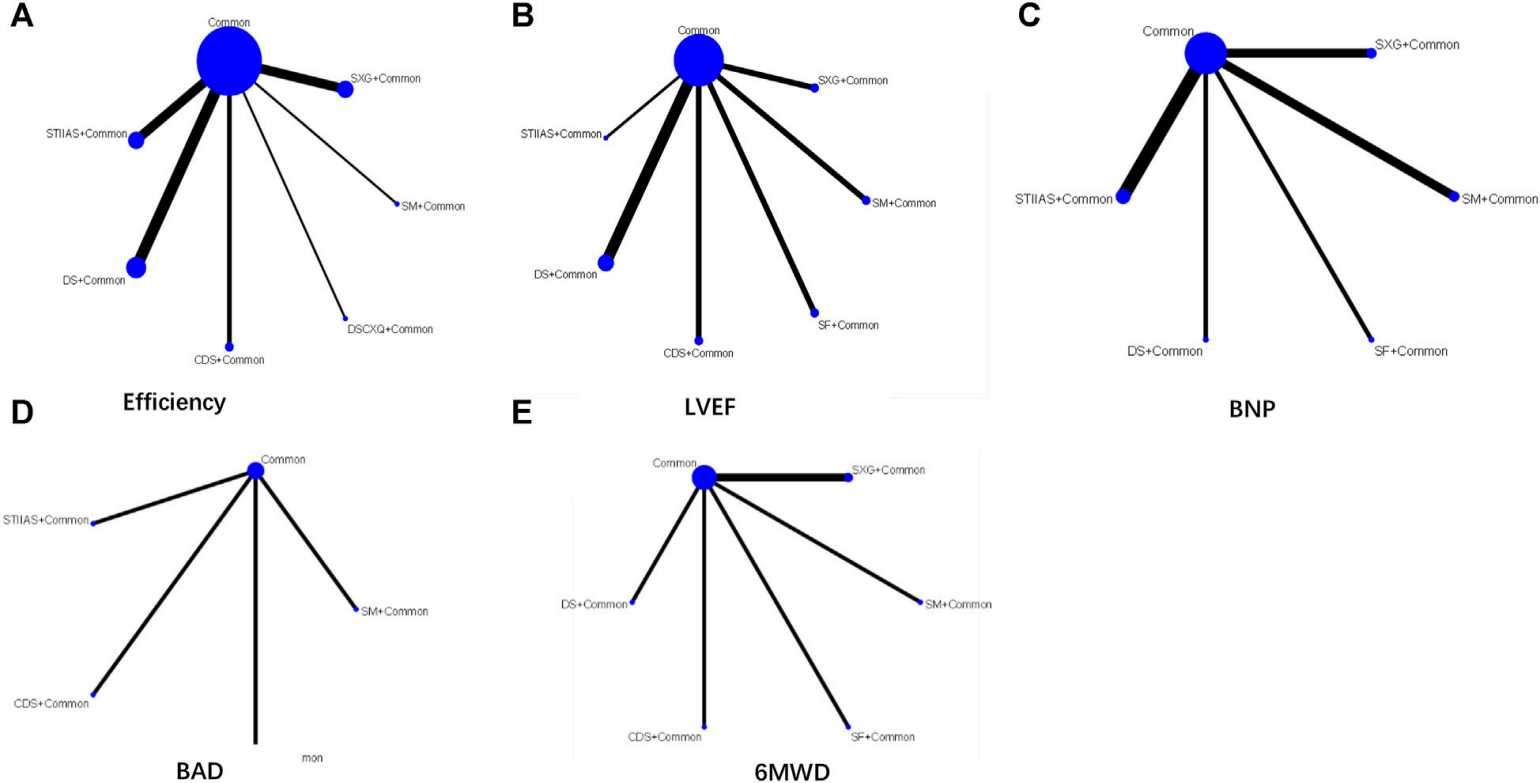
Network maps of comparisons on different outcomes of interventions **(A)** Efficiency **(B)** LVEF **(C)** BNP **(D)** BAD **(E)** 6MWD. The size of the nodes relates to the number of participants in that intervention type. And the thickness of lines between the interventions relates to the number of studies for that comparison. SFI, Shenfu injection; SMI, Shengmai injection; SXG, Shenxiong glucose injection; STIIAS, Sodium tanshinone IIA sulfonate injection; DS, Danshen injection; CDS, Compound Danshen injection; SF, Shenfu Injection; DSCXQ, Danshenchuanxiongqin injection.

In the 24 included studies, 7 different interventions were involved, including Shengmai Injection + Common, Shenxiong Glucose Injection + Common, Sodium Tanshinone IIA Sulfonate Injection + Common, Danshen injection + Common, Compound Danshen injection + Common, Shenfu Injection + Common, and Danshenchuanxiongqin Injection. The network of various interventions for each outcome indicator is shown in Figure 3. In the figure, the thickness of the lines is proportional to the amount of literature compared between the two, and the size of the diameter of the circles is proportional to the number of participants involved in the intervention.

#### 3.4.2 Total EF

**FIGURE 4:**
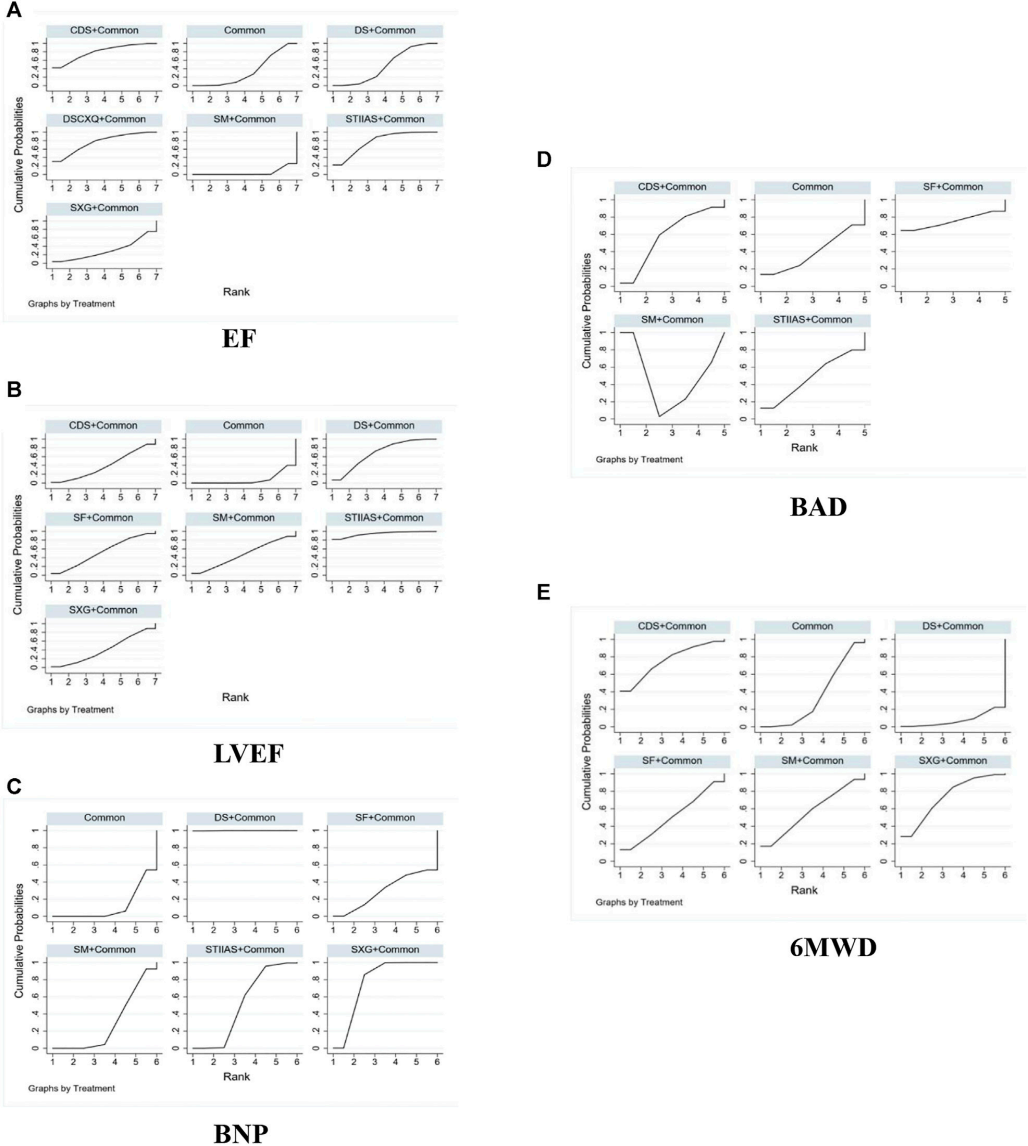
SUCRA of comparisons on different outcomes of interventions. **(A)** EF **(B)** LVEF **(C)** BNP **(D)** BAD **(E)** 6NWD. Notes: In this graphical approach, rankings are presented through examining the area under the curve. The bigger the area under the curve, the higher the likelihood that an intervention is in the top rank or one of the top ranks. SFI, Shenfu injection; SMI, Shengmai injection; SXG, Shenxiong glucose injection; STIIAS, Sodium tanshinone IIA sulfonate injection; DS, Danshen injection; CDS, Compound Danshen injection; SF, Shenfu Injection; DSCXQ, Danshenchuanxiongqin injection.

**FIGURE 5:**
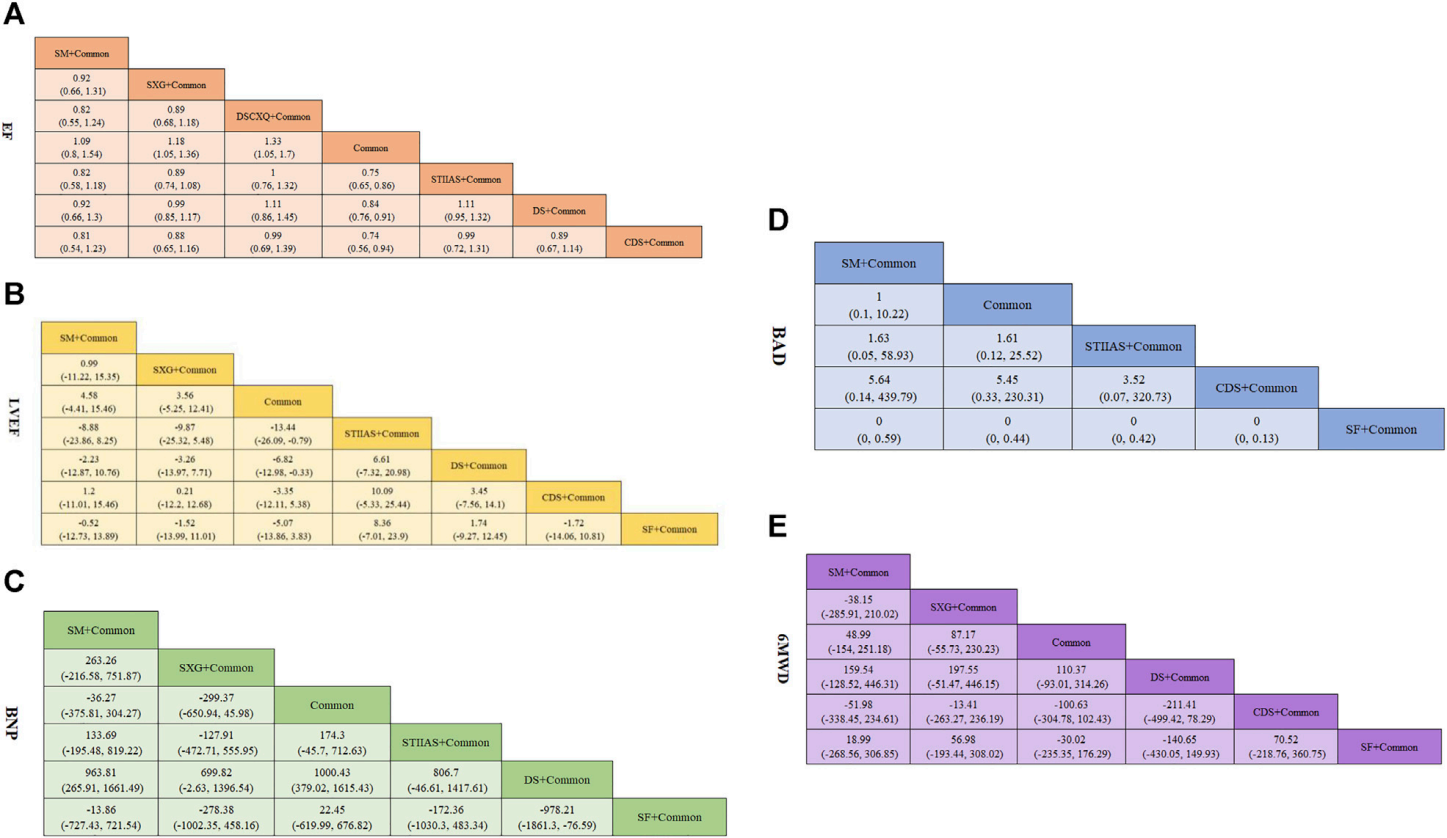
Pooled estimates of the network meta-analysis. **(A)** EF **(B)** LVEF **(C)** BNP **(D)** BAD **(E)** 6XLWD. Estimates are presented as column *versus* row for the network meta-analyses to make network and pairwise meta-analysis results directly comparable. Effect estimates are presented as pooled WAD or RR with 95% CIs. SFI, Shenfu injection; SMI, Shengmai injection; SXG, Shenxiong glucose injection; STIIAS, Sodium tanshinone IIA sulfonate injection; DS, Danshen injection; CDS, Compound Danshen injection; SF, Shenfu Injection; DSCXQ, Danshenchuanxiongqin injection.

The indicator of total EF was reported in 17 studies (Hu, 2012; Xin and Shao, 2012; Yan, 2012; Tang, 2013; Cao, 2015; Hu, 2015; Wang and Gao, 2015; Zeng et al., 2015; Zhang et al., 2015; Jiang et al., 2016; Li, 2016; Lyu et al., 2016; Qin and Jiang, 2016; Qiao, 2017; Yao, 2018; Ou and Wang, 2019; Shang and Lyu, 2019). The evidence map is shown in Figure 3A. Outcomes were presented as risk ratios (RR) and the corresponding 95% ci were reported. A statistically significant difference was considered to be present if the 95% CI did not include the value “1”. Specially, when the point estimate and confidence interval were >1, the total EF in the test group was considered superior to that in the conventional western medicine group. Conversely, when the point estimate and confidence interval were <1, the total EF in the test group was considered inferior to that in the conventional western medicine group. Shengmai Injection + Common (RR = 1.1; 95% CI: 0.8, 1.54), Shenxiong Glucose Injection + Common (RR = 1.19; 95% CI: 1.06, 1.37), Danshenchuanxiongqin Injection + Common (RR = 1.33; 95% CI: 1.05, 1.7), Sodium Tanshinone IIA Sulfonate Injection + Common (RR = 1.33; 95% CI: 1.17, 1.54), Danshen injection + Common (RR = 1.19; 95% CI: 1.09, 1.31), and Compound Danshen injection + Common (RR = 1.35; 95% CI: 1.05, 1.77) may be superior to common Western medicine treatment alone in improving EF, and the difference was statistically significant (*p* < 0.05), as shown in Figure 5A. In the ranking of interventions, Compound Danshen injection + Common (SUCRA: 79.6%), Sodium Tanshinone IIA Sulfonate Injection + Common (SUCRA: 78.0%), and Common + Danshenchuanxiongqin Injection (SUCRA: 76.0%) could achieve the optimal effects in improving EF. The ranked probability is shown in Figure 4A.

#### 3.4.3 LVEF

In 13 studies (Li, 2011; Hu, 2012; Xin and Shao, 2012; Cao, 2015; Wang and Gao, 2015; Zeng et al., 2015; Lyu et al., 2016; Xian et al., 2016; Li et al., 2018; Ou and Wang, 2019; Shang and Lyu, 2019; Wang et al., 2019; Huang et al., 2022), the indicator of LVEF was reported, and the changes in LVEF after treatment with Danshen injections were compared. The evidence map is shown in Figure 3B. Outcomes were presented as risk ratios (RR) and the corresponding 95% ci were reported. A statistically significant difference was considered to be present if the 95% CI did not include the value “1”. Specially, when the point estimate and confidence interval were >1, LVEF was deemed superior in the test group compared to the conventional western medicine group. Conversely, when the point estimate and confidence interval were <1, LVEF was considered lower in the test group than in the conventional western medicine group. Based on the NMA results, SM + Common (WMD = 4.58, 95% CI: −4.41, 15.46), SXG + Common (WMD = 3.56, 95% CI: −5.25, 12.41), STIIAS + Common (WMD = 13.44, 95% CI: 0.79, 26.09), DS + Common (WMD = 6.82, 95% CI: 0.33, 12.98), and CDS + Common (WMD = 3.35, 95% CI: −5.38, 12.11) outperformed common Western medicine treatment alone in improving EF, and the difference was statistically significant (*p* < 0.05), as shown in Figure 5B (league table). In the ranking of interventions, Sodium Tanshinone IIA Sulfonate Injection + Common (SUCRA: 94.3%), Danshen injection + Common (SUCRA: 68.2%), Shenfu Injection + Common (SUCRA: 52.7%) were the most effective in improving LVEF (*p* < 0.05). The ranked probability is shown in Figure 4B.

#### 3.4.4 BNP

In 9 studies (Jiang et al., 2016; Lyu et al., 2016; Qin and Jiang, 2016; Xian et al., 2016; Qiao, 2017; Yao, 2018; Wang et al., 2020; Li et al., 2022b; Huang et al., 2022), the indicator of BNP was reported, and the changes in BNP after treatment with Danshen injections were compared. The evidence map is shown in Figure 3C. Outcomes were presented as risk ratios (RR) and the corresponding 95% ci were reported. A statistically significant difference was considered to be present if the 95% CI did not include the value “1.” Specially, the trial group was regarded as more effective for BNP than the conventional western medicine group when the point estimate and confidence interval were >1. Conversely, the trial group was considered less effective for BNP than the conventional western medicine group when the point estimate and confidence interval was <1. Based on the NMA results, SM + Common (WMD = −36.27, 95% CI: −375.81, 304.27), SXG + Common (WMD = −299.37, 95% CI: −650.94, 45.98), STIIAS + Common (WMD = −174.3, 95% CI: −712.63, 45.7), DS + Common (WMD = −1,000.43, 95% CI: −1,615.43, −379.02), and SF + Common (WMD = −22.45, 95% CI: −676.82, 619.99) were more effective than common Western treatment alone in improving BNP, and the difference was statistically significant (*p* < 0.05), as shown in Figure 5C (league table). In the ranking of interventions, Danshen injection + Common (SUCRA: 99.9%), Shenxiong Glucose Injection + Common (SUCRA: 77.2%), and Sodium Tanshinone IIA Sulfonate Injection + Common (SUCRA: 51.5%) could achieve the optimal effects in improving BNP. The ranked probability is shown in Figure 4C.

#### 3.4.5 Adverse reactions

In 4 studies (Cao, 2015; Lyu et al., 2016; Xian et al., 2016; Wang et al., 2019), the indicator of adverse reactions was reported, and the safety of Danshen injections in cardiac failure was compared. The evidence map is shown in Figure 3D. Outcomes were presented as risk ratios (RR) and the corresponding 95% ci were reported. A statistically significant difference was considered to be present if the 95% CI did not include the value “1”. Specially, when the point estimates and confidence intervals were >1, the risk of adverse events in the trial group was considered to be higher and thus more dangerous, and conversely, when the point estimates and confidence intervals were <1, the risk of adverse events in the trial group was considered to be safer compared to the conventional Western medicine group. Based on the NMA results, SM + Common (RR = 1, 95% CI: 0.1, 10.22), STIIAS + Common (RR = 0.62, 95% CI: 0.04, 8.62), CDS + Common (RR = 0.18, 95% CI: 0, 0.36), and SF + Common (RR = 310,334.94, 95% CI: 2.26, 30598537594444904) were superior to common Western treatment alone in improving adverse reactions, and the difference was statistically significant (*p* < 0.05), as shown in Figure 5D (league table). In the intervention ranking, Shenfu Injection + Common (SUCRA: 75.2%), Compound Danshen injection + Common (SUCRA: 58.8%), and Sodium Tanshinone IIA Sulfonate Injection + Common (SUCRA: 48.5%) were the three intervention regimens with the lowest risk of adverse reactions (*p* < 0.05). The ranked probability is shown in Figure 4D adverse reactions included four cases of gastrointestinal reactions, one case of respiratory system injury, and one case of severe adverse reactions induced by acute appendicitis. Other adverse reactions could be spontaneously relieved after drug withdrawal or symptomatic treatment. This suggests that Danshen injections + Common in cardiac failure would not increase adverse reactions, indicating a good safety profile of Danshen injections.

#### 3.4.6 6MWD

Six studies (Wang and Gao, 2015; Zeng et al., 2015; Xian et al., 2016; Ou and Wang, 2019; Wang et al., 2019; Huang et al., 2022) reported 6MWD. The evidence map is shown in Figure 3E. Outcomes were presented as risk ratios (RR) and the corresponding 95% ci were reported. A statistically significant difference was considered to be present if the 95% CI did not include the value “1.” Specially, the 6MWD in the test group was considered to be higher than that in the conventional western medicine group when the point estimate and confidence interval were >1. Conversely, the 6MWD in the test group was considered to be lower than that in the conventional western medicine group when the point estimate and confidence interval was <1. Based on the NMA results, SM + Common (WMD = 48.99, 95% CI: −154, 251.18), SXG + Common (WMD = 87.17, 95% CI: −55.73, 230.23), DS + Common (WMD = −110.37, 95% CI: −314.26, 93.01), CDS + Common (WMD = 100.63, 95% CI: −102.43, 304.78), and SF + Common (WMD = 30.02, 95% CI: −176.29, 235.35) were superior to common Western treatment in improving 6MWD, and the difference was statistically significant (*p* < 0.05), as shown in Figure 5E (league table). In the ranking of interventions, Compound Danshen injection + Common (SUCRA: 75.6%), Shenxiong Glucose Injection + Common (SUCRA: 73.7%), and Shengmai Injection + Common (SUCRA: 57.2%) could achieve the optimal effects in improving 6MWD. The ranked probability is shown in Figure 4E.

## 4 Discussion

### 4.1 Main findings

This paper is the first systematic retrospective analysis of the efficacy and safety of Danshen injection in the treatment of heart failure. Our findings provide an update to the Efficacy and safety of Danshen injection in the treatment of heart failure: a systematic evaluation and meta-analysis protocol (Yuan et al., 2019). Moreover, our results align with a previous study conducted by Mingxuan Li et al. This study, encompassing a total of 148 studies with 14,979 patients and evaluating eight Danshen-based injections-namely, Danshen injection, Compound Danshen injection, Coronary Heart injection, Danshen Chuanxiongzizine injection, Danhong injection, Danshentong IIA xanthic acid injection, Ginsengxiong Grapeshot injection, and Danshen Dofenacitis injection, and it was concluded that all of the eight danshen-based injections were effective (Li et al., 2022a), Cardiac failure is the most severe and late phase of various heart diseases, with a high mortality and readmission rate. The mortality of patients in the intensive care unit (ICU) from cardiac failure has reached 4.1% (Li et al., 2021), with the symptoms manifested as peripheral edema, fluid retention, and dyspnea (Yancy et al., 2013; Wang and Liang, 2018). To the best of our knowledge, this is the first network meta-analysis (NMA) comparing the effectiveness and safety of different Danshen injections in combination with common Western treatment for heart failure. The NMA has revealed that Danshen injections + Common can effectively alleviate various symptoms of patients with cardiac failure. Specifically, Compound Danshen injection + Common is the most effective measure to improve the total EF; Sodium Tanshinone IIA Sulfonate Injection + Common is the most effective measure to improve LVEF; Danshen injection + Common is the most effective measure to improve BNP; Shenfu Injection + Common is the most effective measure to improve adverse reactions, and Compound Danshen injection + Common is the most effective measure to improve 6MWD. Our findings are consistent with the results of a previous meta-analysis (Bai et al., 2018) that included 24 papers involving five injections, including Shenfu Injection, Shenmai Injection, Shengmai Injection, Danhong Injection, and Astragalus Injection. That meta-analysis revealed that Danshen injections + Common could effectively improve relevant symptoms, and Shenfu Injection, Shenmai Injection, and Shengmai Injection could significantly improve 6MWD and total EF. A recent pharmacological study has shown that the effective ingredient of Danshen can significantly reduce blood viscosity, protect the cardiovascular system, and alleviate myocardial hypoxia injury to significantly decrease the ventricular hypertrophy index, thus improving the ventricular myocardial function and regulating the ventricular diastolic function (Yuan et al., 2019).

In terms of total EF, Danshen injection + Common is superior to Western treatment alone, and Compound Danshen injection + Common can achieve the most significant effect. Compound Danshen injection + Common is believed to improve the effectiveness of Western medicine treatment for cardiac failure as adjuvant therapy. A previous study (Bian et al., 2021) has revealed that Rosuvastatin + Compound Danshen Injection is effective in the treatment of acute cerebral infarction, which can effectively improve the clinical symptoms of patients, reduce the incidence of complications, and promote the recovery of IL-6, CRP, and D-dimer levels. Another study (Liu et al., 2019) shows that Compound Danshen injection + Butylphthalide Injection can achieve good clinical effectiveness in old patients with acute cerebral infarction.

In terms of improving LVEF, Sodium Tanshinone IIA Sulfonate Injection + Common is the most effective measure. A previous meta-analysis included 14 RCTs involving 1,368 patients and employed the RevMan 5.3 software for data analysis (Shao et al., 2022). The results revealed that Sodium Tanshinone IIA Sulfonate Injection appeared to be more effective than Western treatment alone in cardiac failure as adjuvant therapy. Specifically, the results of the meta-analysis of LVEF showed that Sodium Tanshinone IIA Sulfonate Injection could reduce high-sensitivity C-reactive protein (hs-CRP) and was independently related to cardiovascular events coronary artery disease (CAD). Reduced hs-CRP levels could further decrease the incidence of cardiac failure.

In terms of improving BNP, Danshen injection + Common is the most effective measure, which is consistent with the results of the study (Shao et al., 2018). In the previous study, 10 RCTs were analyzed, involving 944 patients with angina pectoris. It was concluded that Danshen injection + antianginal drugs could improve the angina pectoris symptoms compared with antianginal drugs alone (beta blockers, calcium antagonists, nitrates, *etc.*). Our NMA has also demonstrated the effectiveness of Danshen injection + Common in patients with cardiac failure.

In terms of adverse reactions, Shenfu Injection + Common is the safest measure. A previous study (Wang et al., 2009) has demonstrated that Shenfu Injection has an obvious protective effect on inducing apoptosis of injured myocardial cells by inhibiting the downregulation of Bcl2-2 protein and the sequential activation of caspase-3, thus proving the safety of Shenfu Injection.

Impaired motion ability is the main symptom of cardiac failure. 6MWD is a common technique to measure motion ability, which can provide useful prognostic information for all-cause hospitalization and mortality of various chronic diseases such as cardiac failure. Studies have shown that 6MWD is not only closely related to the peak aerobic capacity of patients with heart disease but also associated with decreases in several traditional indicators of the left ventricle, left atrium, and right ventricle dysfunction (Chen et al., 2016; Patel et al., 2021). Based on our meta-analysis results, Compound Danshen injection + Common is the most effective measure to improve 6MWD. An animal experiment has revealed that Compound Danshen injection can significantly reduce the infarct area, inhibit the expression of caspase-3 protein in myocardial tissue, and lower the level of MDA and the activities of CK, LDH, and cTnI in serum. By activating the Akt-eNOS signaling pathway, Compound Danshen injection can protect myocardial cells from MI/R injury, inhibit myocardial cell apoptosis, and further reduce the incidence of cardiac failure (Ren-an et al., 2014). Regulating cardiac systolic function and reducing cardiac load can promote the recovery of motor function. The results are similar to the findings of our NMA.

### 4.2 Limitations

Even though our study has fully demonstrated the advantages of Danshen injections in cardiac failure, there are still three limitations. Firstly, the quality of many included RCTs was relatively low. To reduce the selection bias, good randomization methods should be used in the included RCTs. In our study, however, only half of the RCTs described the appropriate randomization methods. Secondly, the blinding methods for investigators or patients were not described in all RCTs. These shortcomings may lead to overestimation or underestimation of the effectiveness of Danshen injections. Thirdly, the results of some RCTs may be inaccurate due to short treatment times and small sample sizes. Therefore, RCTs with more rigorous design and higher quality are still required.

## 5 Conclusion

In our study, six types of commonly used Danshen injections were assessed based on different outcome indicators, and the effectiveness of these Danshen injections was ranked. The NMA shows that Compound Danshen injection has the highest EF compared with Western medicine treatment. Danshen injection + Common can achieve the optimal effect in reducing BNP, while Compound Danshen injection + Common can achieve the optimal effect in improving the total EF and 6MWD. In terms of improving LVEF, Sodium Tanshinone IIA Sulfonate Injection + Common can achieve the optimal effect, while Shenfu Injection + Common has the highest safety. Although the current study has demonstrated the unique advantages and effectiveness of TCM in cardiac failure, the safety, effectiveness, and standardization of its clinical application still need further investigation. In the future, more high-quality RCTs with better trial designs are desired to distinguish the degree of patients’ illness, unify the dosage of medication, and focus on more specific safety indicators, in the hope of providing more targeted and effective treatments for patients with cardiac failure. Endeavors should be made to promote the standardization and application of Danshen injections.
