# Supplementary material for: Effectiveness and safety of Danshen injections in treatment of cardiac failure: a network meta-analysis
Source: Front Pharmacol. 2024 Mar 13;15:1319551. doi: 10.3389/fphar.2024.1319551 (PMC10966466; doi:10.3389/fphar.2024.1319551)
Supplement: Supplementary file 1 [file Table1.DOCX]

# Table S1 Literature search strategy

**1.Pubmed**

| Search number | Query |
| --- | --- |
| #1 | "Heart Failure"[Mesh] |
| #2 | (((((Heart Failure[Title/Abstract]) OR (Cardiac Failure[Title/Abstract])) OR (Heart Decompensation[Title/Abstract])) OR (Myocardial Failure[Title/Abstract])) OR (cardiac decompensation[Title/Abstract])) OR (myocardial failure[Title/Abstract]) |
| #3 | ("Heart Failure"[Mesh]) OR ((((((Heart Failure[Title/Abstract]) OR (Cardiac Failure[Title/Abstract])) OR (Heart Decompensation[Title/Abstract])) OR (Myocardial Failure[Title/Abstract])) OR (cardiac decompensation[Title/Abstract])) OR (myocardial failure[Title/Abstract])) |
| #4 | "Medicine, Chinese Traditional"[Mesh] |
| #5 | "Salvia miltiorrhiza"[Mesh] |
| #6 | ((((traditional Chinse medicine[Title/Abstract]) OR (TCM[Title/Abstract])) OR (Danshen injection[Title/Abstract])) OR (Danhong injection[Title/Abstract])) OR (Salvia miltiorrhiza[Title/Abstract]) |
| #7 | (("Medicine, Chinese Traditional"[Mesh]) OR ("Salvia miltiorrhiza"[Mesh])) OR (((((traditional Chinse medicine[Title/Abstract]) OR (TCM[Title/Abstract])) OR (Danshen injection[Title/Abstract])) OR (Danhong injection[Title/Abstract])) OR (Salvia miltiorrhiza[Title/Abstract])) |
| #8 | "Randomized Controlled Trial" [Publication Type] |
| #9 | ((((randomized controlled trial[Title/Abstract]) OR (randomised controlled study[Title/Abstract])) OR (clinical trial[Title/Abstract])) OR (RCT[Title/Abstract])) OR (random*[Title/Abstract]) |
| #10 | ("Randomized Controlled Trial" [Publication Type]) OR (((((randomized controlled trial[Title/Abstract]) OR (randomised controlled study[Title/Abstract])) OR (clinical trial[Title/Abstract])) OR (RCT[Title/Abstract])) OR (random*[Title/Abstract])) |
| #11 | ((("Heart Failure"[Mesh]) OR ((((((Heart Failure[Title/Abstract]) OR (Cardiac Failure[Title/Abstract])) OR (Heart Decompensation[Title/Abstract])) OR (Myocardial Failure[Title/Abstract])) OR (cardiac decompensation[Title/Abstract])) OR (myocardial failure[Title/Abstract]))) AND ((("Medicine, Chinese Traditional"[Mesh]) OR ("Salvia miltiorrhiza"[Mesh])) OR (((((traditional Chinse medicine[Title/Abstract]) OR (TCM[Title/Abstract])) OR (Danshen injection[Title/Abstract])) OR (Danhong injection[Title/Abstract])) OR (Salvia miltiorrhiza[Title/Abstract])))) AND (("Randomized Controlled Trial" [Publication Type]) OR (((((randomized controlled trial[Title/Abstract]) OR (randomised controlled study[Title/Abstract])) OR (clinical trial[Title/Abstract])) OR (RCT[Title/Abstract])) OR (random*[Title/Abstract]))) |

**2.Cochrane**

| Search number | Query |
| --- | --- |
| #1 | MeSH descriptor: [Heart Failure] explode all trees |
| #2 | (Heart Failure):ti,ab,kw OR (Cardiac Failure):ti,ab,kw OR (Heart Decompensation):ti,ab,kw OR (Myocardial Failure):ti,ab,kw OR (cardiac decompensation):ti,ab,kw |
| #3 | (myocardial failure):ti,ab,kw |
| #4 | #1 or #2 or #3 |
| #5 | MeSH descriptor: [Medicine, Chinese Traditional] explode all trees |
| #6 | MeSH descriptor: [Salvia miltiorrhiza] explode all trees |
| #7 | (traditional Chinse medicine):ti,ab,kw OR (TCM):ti,ab,kw OR (Danshen injection):ti,ab,kw OR (Danhong injection):ti,ab,kw OR (Salvia miltiorrhiza):ti,ab,kw |
| #8 | #5 or #6 or #7 |
| #9 | MeSH descriptor: [Randomized Controlled Trial] explode all trees |
| #10 | (randomized controlled trial):ti,ab,kw OR (randomised controlled study):ti,ab,kw OR (clinical trial):ti,ab,kw OR (RCT):ti,ab,kw OR (random*):ti,ab,kw |
| #11 | #9 or #10 |
| #12 | #4 and #8 and #11 |

**3.Embase**

| Search number | Query |
| --- | --- |
| #1 | 'heart failure'/exp |
| #2 | 'heart failure':ab,ti OR 'cardiac failure':ab,ti OR 'heart decompensation':ab,ti OR 'cardiac decompensation':ab,ti OR 'myocardial failure':ab,ti |
| #3 | #1 OR #2 |
| #4 | 'chinese medicine'/exp |
| #5 | 'salvia miltiorrhiza'/exp |
| #6 | 'traditional chinse medicine':ab,ti OR tcm:ab,ti OR 'danshen injection':ab,ti OR 'danhong injection':ab,ti OR 'salvia miltiorrhiza':ab,ti |
| #7 | #4 OR #5 OR #6 |
| #8 | 'randomized controlled trial'/exp |
| #9 | 'randomized controlled trial':ab,ti OR 'randomised controlled study':ab,ti OR 'clinical trial':ab,ti OR rct:ab,ti OR random*:ab,ti |
| #10 | #8 OR #9 |
| #11 | #3 AND #7 AND #10 |

**4.Web of science**

| Search number | Query |
| --- | --- |
| #1 | Heart Failure (Topic) OR Cardiac Failure (Topic) OR Heart Decompensation (Topic) OR Myocardial Failure (Topic) OR cardiac decompensation (Topic) OR myocardial failure (Topic) |
| #2 | traditional Chinse medicine (Topic) OR TCM (Topic) OR Danshen injection (Topic) OR Danhong injection (Topic) OR Salvia miltiorrhiza (Topic) |
| #3 | randomized controlled trial (Topic) OR randomised controlled study (Topic) OR clinical trial (Topic) OR RCT (Topic) OR random* (Topic) |
| #4 | #3 AND #2 AND #1 |

**5. The following table shows an example of a search strategy for Chinese databases (Sinomed).**

| Search number | Query |
| --- | --- |
| #1 | "心力衰竭"[不加权:扩展] |
| #2 | ( "心力衰竭"[常用字段:智能] OR "心衰"[常用字段:智能]) |
| #3 | (( "心力衰竭"[常用字段:智能] OR "心衰"[常用字段:智能])) OR ("心力衰竭"[不加权:扩展]) |
| #4 | "丹参注射液"[不加权:扩展] |
| #5 | ( "丹参注射剂"[常用字段:智能] OR "丹参川芎嗪注射剂"[常用字段:智能] OR "丹参多酚酸注射剂"[常用字段:智能] OR "复方丹参注射剂"[常用字段:智能] OR "丹参多酚酸注射剂"[常用字段:智能] OR "丹参多酚酸盐注射剂"[常用字段:智能] OR "丹参酮ⅡA磺酸钠注射剂"[常用字段:智能] OR "丹红注射剂"[常用字段:智能] OR "参芎葡萄糖注射剂"[常用字段:智能]) |
| #6 | (( "丹参注射剂"[常用字段:智能] OR "丹参川芎嗪注射剂"[常用字段:智能] OR "丹参多酚酸注射剂"[常用字段:智能] OR "复方丹参注射剂"[常用字段:智能] OR "丹参多酚酸注射剂"[常用字段:智能] OR "丹参多酚酸盐注射剂"[常用字段:智能] OR "丹参酮ⅡA磺酸钠注射剂"[常用字段:智能] OR "丹红注射剂"[常用字段:智能] OR "参芎葡萄糖注射剂"[常用字段:智能])) OR ("丹参注射液"[不加权:扩展]) |
| #7 | ((( "丹参注射剂"[常用字段:智能] OR "丹参川芎嗪注射剂"[常用字段:智能] OR "丹参多酚酸注射剂"[常用字段:智能] OR "复方丹参注射剂"[常用字段:智能] OR "丹参多酚酸注射剂"[常用字段:智能] OR "丹参多酚酸盐注射剂"[常用字段:智能] OR "丹参酮ⅡA磺酸钠注射剂"[常用字段:智能] OR "丹红注射剂"[常用字段:智能] OR "参芎葡萄糖注射剂"[常用字段:智能])) OR ("丹参注射液"[不加权:扩展])) AND ((( "心力衰竭"[常用字段:智能] OR "心衰"[常用字段:智能])) OR ("心力衰竭"[不加权:扩展])) |
